# Screening of In Vitro Health Benefits of Tangerine Tomatoes

**DOI:** 10.3390/antiox8070230

**Published:** 2019-07-19

**Authors:** Hartono Tanambell, Siew Young Quek, Karen Suzanne Bishop

**Affiliations:** 1 Food Science, School of Chemical Sciences, The University of Auckland, Private Bag 92019, Auckland 1142, New Zealand; 2 Auckland Cancer Society Research Centre, School of Medical Sciences, Faculty of Medicine and Health Sciences, The University of Auckland, Private Bag 92019, Auckland 1142, New Zealand; 3 Riddet Institute, New Zealand Centre of Research Excellence for Food Research, Palmerston North 4474, New Zealand; 4 Discipline of Nutrition and Dietetics, School of Medical Sciences, Faculty of Medicine and Health Sciences, The University of Auckland, Private Bag 92019, Auckland 1142, New Zealand

**Keywords:** Anti-inflammation, antioxidant, in vitro, lycopene isomers, prostate cancer, tomatoes, tomato extracts

## Abstract

Tomatoes have been associated with various health benefits, including the prevention of chronic diseases. The *cis*-isomers of lycopene occurring in tangerine tomatoes were, through clinical trials, proven to be more bioavailable than the all-*trans* lycopene found in red tomatoes. Nonetheless, scientific evidence regarding the bioactivities of the tangerine tomatoes is lacking. In this article, the antioxidant, anticancer, and anti-inflammatory properties of extracts prepared from four different tomato varieties, namely Alfred, Olga’s Round Golden Chicken Egg, Golden Green, and Golden Eye, were investigated. While the antioxidant capacities of the extracts were measured through the ferric reducing antioxidant power (FRAP) and 2,2’-azino-bis(3-ethylbenzothiazoline-6-sulphonic acid) (ABTS) assays, their anti-proliferative properties in prostate cancer cell lines were examined through the Sulforhodamine-B (SRB) assay. The anti-inflammatory activities of the extracts were assessed through the toll-like receptor (TLR)2, TLR4, and nucleotide-binding oligomerization domain containing protein 2 (NOD2)-mediated inflammatory pathways. Our results show that the tangerine tomatoes had lower IC50 values in both the anticancer and anti-inflammatory assays compared to the red tomatoes. Specifically, the half-maximal inhibitory concentration (IC50) values of the tangerine tomatoes in LNCaP cells were approximately two to three fold lower than the red tomato (IC50: 14.46, 5.62, and 8.08 mg dry tomato equivalent/mL from Alfred hexane-acetone, Olga’s Round Golden Chicken Egg hexane, and Golden Green hexane, respectively). These findings indicate that the tangerine varieties, Olga’s Round Golden Chicken Egg and Golden Green, possess greater potential to be used in conjunction with treatment and for the prevention of cancer and inflammatory-related diseases than the Alfred (red) and Golden Eye (high beta-carotene) varieties.

## 1. Introduction

The consumption of tomatoes and tomato-based products has been suggested to minimize the risks of cardiovascular disease [1] and several types of cancer [2,3,4]. These health benefits are thought to be attributed to the phytonutrient, lycopene, the principal carotenoid of tomatoes [5,6]. Lycopene predominantly occurs in the all-*trans* form in unprocessed red tomatoes, and in *cis*, particularly the tetra-*cis* isomer in tangerine tomatoes [7]. These isomers naturally occur in different matrixes; tetra-*cis* lycopene is found in lipid-soluble globular matrixes in tangerine tomatoes, while all-*trans* lycopene is deposited in large crystalline aggregates in red tomatoes [7]. The difference between the matrixes is thought to be attributed to the structural bending of the *cis*-isomers [8]. 

Based on clinical trials, the *cis* isomers of lycopene were more bioavailable in humans than the all-*trans* counterpart [7,8,9]. This phenomenon is likely to be linked to the absorption process of lycopene within the human body [10]. In the human body, lycopene is taken up by the mucosa of the small intestine, and subsequently transported in chylomicrons to the liver or other organs via the bloodstream [11]. It is hypothesized that the absorption of lycopene by the mucosa of the small intestine is aided by the formation of bile acid micelles [10]. Therefore, the higher bioavailability of -*cis*-isomers may be attributed to its better solubility in the bile acid micelles [10]. Additionally, the more bioavailable nature of the *cis*-isomer might also be related to the abovementioned difference in the matrix of deposition [7,8]. 

Correspondingly, tangerine tomatoes may exert greater health benefits than red tomatoes. However, it should be noted that most, if not all studies on the potential health benefits of tomatoes were performed using red tomatoes. Furthermore, it is worth mentioning that the reason tangerine tomatoes accumulate tetra-*cis* instead of all-*trans* lycopene is that it lacks the enzyme carotenoid isomerase, which converts poly-*cis* into all-*trans* lycopene [12]. Consequently, it is unlikely for both all-*trans* and tetra-*cis* lycopene to simultaneously occur in significant amounts in the same tomato variety. Therefore, it is unknown whether tangerine tomatoes containing tetra-*cis* lycopene possess greater health benefits than red tomatoes containing all-*trans* lycopene. Grounded on this scientific gap, in this study we screened the potential in vitro health benefits of some tangerine tomato extracts, and compared them to that of red tomato extracts. 

In addition to the red tomatoes, our study also included the high-beta carotene tomatoes as another source of comparison. The high-beta carotene tomatoes were included in the study because they are orange-colored tomatoes that accumulate beta-carotene instead of tetra-cis lycopene. Since the high-beta carotene and tangerine varieties are phenotypically indistinguishable, it is important to determine whether both varieties exert similar health benefits.

Prostate cancer (PCa) is one of the cancers with an incidence rate that is reported to be inversely associated with consumption of tomatoes and tomato-based products [2]. Although the mechanism of how tomatoes or lycopene prevent PCa has not been robustly defined, it has been reported that lycopene suppresses inflammation, proliferation of prostate epithelial cells, and oxidative DNA damage [13]. The relationship between these properties and cancer can be explained through the hallmarks of cancer [14]. Inflammation is known as an enabling characteristic of cancer which governs various pathways underlying cancer [14]. Moreover, cell proliferation is a hallmark of cancer [14]. Additionally, although oxidation is not a cancer hallmark, it is known to cause DNA damage [13], which eventually leads to the cancer enabling characteristics of genomic instability [14]. 

Supplementary to the abovementioned arguments, PCa has been reported to be in the top two most prevalent cancers amongst men in the world [15], and the most common cancer among men in New Zealand [16]. It accounted for 27.07% of new cancer registrations amongst male New Zealanders in 2016 [16]. Correspondingly, we assessed the effect of the tomato extracts on the in vitro proliferation of PCa cells. In addition, we screened the in vitro antioxidant and anti-inflammatory activities of the tomato extracts as parameters that are related to PCa. 

Our research reports the antioxidant, anti-proliferative, and anti-inflammatory activities of tomato extracts prepared from four different varieties, namely Alfred, Olga’s Round Golden Chicken Egg, Golden Green, and Golden Eye. Alfred was selected because it is a commercial red tomato variety grown and sold locally. Olga’s Round Golden Chicken Egg and Golden Green were selected because they are tangerine tomatoes, while Golden Eye was selected because it is from the high-beta carotene variety. Ultimately, our hypothesis was that tangerine tomatoes might have greater in vitro health benefits than the red and high beta-carotene tomatoes. 

## 2. Materials and Methods 

### 2.1. Materials

All the chemicals used in the extraction, antioxidant analyses, and cell proliferation assays were of analytical grade, unless stated otherwise. The solvents used for extraction were hexane (laboratory reagent grade; ECP Ltd., Auckland, New Zealand), ethanol (ECP Ltd., Auckland, New Zealand), acetone (Macron, New Zealand), and dimethyl sulfoxide (DMSO; Sigma-Aldrich®, Auckland, New Zealand). The chemicals required for the antioxidant analyses were 2,2’-azino-bis(3-ethylbenzothiazoline-6-sulphonic acid) (ABTS), potassium persulfate (K_2_S_2_O_8_), 2,4,6-Tris(2-pyridyl)-s-triazine (TPTZ), and ∝-tocopherol from Sigma-Aldrich®, New Zealand; Acetic acid, ferric chloride (FeCl_3_), and sodium acetate (Na_2_CO_3_) from ECP Ltd., New Zealand; Hydrochloric acid (HCl; Avantor, USA). The chemicals used in the PCa proliferation assays were trichloroacetic acid (TCA) and sulforhodamine-B (SRB) from Sigma-Aldrich®, New Zealand; Glacial acetic acid (Emsure®, New Zealand); Tris(hydroxymethyl)aminomethane (Tris; Invitrogen™, USA); Trypan blue dye (Gibco™, New Zealand).

The materials used in the maintenance of cell culture and for the anti-inflammatory assay were of cell culture grade, unless stated otherwise. The chemicals used for cell culture maintenance were trypsin (Sigma-Aldrich®, New Zealand), disodium ethylenediaminetetraacetic acid (EDTA; Gibco™, New Zealand), and phosphate buffered saline (PBS; Sigma-Aldrich®, New Zealand). The reagents used for the anti-inflammatory assays were ibuprofen (Sigma-Aldrich®, New Zealand), phorbol 12-myristate 13-acetate (PMA; InvivoGen, San Diego, CA, USA), Pam3CysSerLys4 (PAM3CSK4; InvivoGen, USA), lipopolysaccharide (LPS; InvivoGen, USA), muramyl dipeptide (MDP; InvivoGen, USA), QUANTI-Blue™ (InvivoGen, USA), and WST-1 (Roche Applied Sciences, Germany).

Pure water required for the experiments was prepared using a Milli-Q purification system (Millipore Corporation, Burlington, MA, USA).

### 2.2. Tomato Varieties and Extracts

Extracts from four tomato varieties, namely Alfred, Olga’s Round Golden Chicken Egg, Golden Green, and Golden Eye were investigated for their impact on health-related activities. The Alfred variety was grown in “Shane’s Greenhouse”, a commercial greenhouse in Wanganui, New Zealand, while the other three varieties were grown in a greenhouse owned by The Heritage Food Crops Research Trust, Wanganui, New Zealand. All four varieties were grown and harvested in the Summer of 2017/2018 (December 2017–February 2018). Upon receipt, the tomato samples were frozen, cut into quarters, and freeze-dried (LabConco, USA) at 0.133 mBar with a temperature of −80 °C. The freeze-dried samples were subsequently ground into powder and stored at −20 °C prior to extraction.

The extraction of the tomatoes was carried out by using four different extraction solvents as seen in Table 1. Briefly, 0.5 g of freeze-dried tomato and 40 mL of solvent were agitated for 30 min by using a magnetic stirrer (IKA Labortechnik, Staufen, Germany). The extracted solution was filtered using filter paper 3HW (Munktell, Finland) in the dark, and subsequently phase separated by the introduction of 2 mL Milli-Q water. The non-polar layer of each filtered extract solution was subsequently centrifuged at 3400× *g* for 10 min using a Heraeus Labofuge 400 centrifuge (Thermoscientific™, Waltham, MA, USA), and concentrated using nitrogen gas in the dark. DMSO was used to dissolve the extracts at different concentrations, depending on the optimized range for each assay. 

### 2.3. Prostate Cancer Cell Lines and Culture Media

Three PCa cell lines, namely LNCaP clone FGC (ATCC® CRL-1435), DU145 (ATCC® HTB-81), and PC3 (ATCC® CRL-1435), were provided by the Auckland Cancer Society Research Centre. The cell lines were provided at low passage numbers (< 10) in cryopreserved form. The cell lines were authenticated by DNA Diagnostics Ltd. through the short tandem repeats method developed by Masters et al. [21], and aliquots stored in liquid nitrogen until required. Furthermore, the cell lines were routinely tested for mycoplasma contamination and confirmed as negative by using the PlasmoTest™ Mycoplasma Detection Kit (InvivoGen, USA). All three cell lines were cultured in Minimum Essential Medium (MEM; Gibco™, USA) supplemented with 5% Fetal Calf Serum (FCS; Moregate Biotech, New Zealand) and 1% Penicillin (Sigma-Aldrich®, USA)/Streptomycin (Sigma-Aldrich®, USA)/Glutamine (Ambion®, Austin, TX, USA) (PSG). The cells were maintained at 37 °C in a humidified incubator (ThermoScientific™, USA), with 5% CO_2_. In addition, the cells were allowed to proliferate and were passaged (when confluent) for a maximum of three months, after which the cells were discarded and cells were revived from the same cryopreserved stock.

### 2.4. Cell Lines and Culture Media for the Anti-Inflammatory Assays

Four cell lines genetically engineered from the Human Embryonic Kidney (HEK)293 cell line were used for the anti-inflammatory assay. Similar to the PCa cell lines, the cell lines were provided at low passage numbers in cryopreserved form. The HEK-Blue™ *human toll-like receptor* (hTLR)2 (catalog code: hkb-htlr2) and HEK-Blue™ hTLR4 (catalog code: hkb-htlr4) cell lines (InvivoGen, USA) were designed to stably express a *nuclear factor kappa-light-chain-enhancer of activated B cells (NF-κB)-inducible secreted embryonic alkaline phosphatase (SEAP)* reporter gene, as well as the *hTLR2* (for HEK-Blue™ hTLR2) or *hTLR4* (for HEK-Blue™ hTLR4) genes. The nucleotide-binding oligomerization domain containing protein (NOD)2-WT and NOD2-G908R cell lines were developed from HEK293T (ATCC® CRL-11268) by Philpott et al. [22] and Folkard et al. [23], respectively. The cells were prepared through co-transfection with an *NF-κB-inducible SEAP* plasmid, and either the *pUNO-hNOD2* for NOD2-WT or its G908R SNP (*pUNO-hNOD2 G908R*) for NOD2-G908R. All four of these cell lines have limited signaling pathways leading to NF-κB activation. Thus, they can only be triggered by their specific receptors (TLR2 for HEK-Blue™ hTLR2, TLR4 for HEK-Blue™ hTLR4, and NOD2 for NOD2-WT and NOD2-G908R).

The HEK-Blue™ hTLR2 and hTLR4 cell lines were maintained in high glucose Dulbecco’s Modified Eagle’s Medium (DMEM, Life Technologies, USA) supplemented with 10% FCS, 1% PSG, and 0.4% HEK-Blue™ selection antibiotics (InvivoGen, USA). The NOD2-WT and NOD2-G908R cell lines were grown in high glucose DMEM supplemented with 10% FCS, 1% PSG, 0.06% Blasticidin (InvivoGen, USA), and 1% Zeocin (InvivoGen, USA). Identical to the PCa cell lines, the cells were maintained in the conditions described in the previous section for a maximum of three months. 

### 2.5. Antioxidant Assays

The ferric reducing antioxidant power (FRAP) and ABTS assays were originally developed by Benzie and Strain [24] and Miller et al. [25], respectively. Both of these assays were modified [26], and this publication serves as the primary source of our modified methods. An Enspire Multimode Reader (PerkinElmer, USA) was used to measure the absorbance of samples in both assays. For each experiment below, a standard curve was prepared by using α-tocopherol as a standard [27]. The results of these experiments were reported as millimol α-tocopherol equivalent per the equivalent of one gram dry tomato (mmol α-tocopherol/g dry tomato equivalent). Each antioxidant assay was performed in two replicates with three technical repeats.

#### 2.5.1. FRAP Assay

The FRAP working solution was prepared by mixing 300 mM acetate buffer pH 3.6, 10 mM TPTZ, and 20 mM FeCl_3_ at a ratio of 10:1:1. Briefly, an amount of 200 μL FRAP working solution was added to 10 μL of sample/blank/standard. The absorbance of the solution was measured at 593 nm, following one hour of incubation in the dark at room temperature. 

#### 2.5.2. ABTS Assay

Solutions of 2.45 mM K_2_S_2_O_8_ and 7 mM ABTS were mixed to prepare the ABTS working solution. Following 12 h incubation of the working solution, its absorbance at 734 nm was adjusted to 0.7 ± 0.01. An amount of 190 μL ABTS working solution was then added to 10 μL of sample, blank, or standard. After incubation in the dark at room temperature for 60 min, the absorbance of the solution was measured at 734 nm.

### 2.6. Prostate Cancer Proliferation Assay

The experiments were carried out under aseptic conditions, i.e. through the use of a class 2 biosafety cabinet (Thermoscientific™, USA) that had been sterilized with ultraviolet light and 70% ethanol prior to and after usage. Each PCa cell line was harvested when 90% confluent, and seeded at a density of 2500 cells/well in 96-well plates. The cells were subsequently incubated overnight as described previously (Section 2.3). All sixteen extracts were then added to the wells at gradient concentrations (0.94–20 mg dry tomato equivalent/mL) through a series of serial dilutions. Following incubation for 96 h, cell proliferation was measured using the SRB assay [28]. A dose-response curve, for each extract and cell line, was then generated using IBM® SPSS® Statistics version 22 (IBM, USA). The dose-response curves were then used for calculating the half-maximal inhibitory concentration (IC50) of the extracts. This experiment was performed for two biological repeats and four technical repeats.

### 2.7. Anti-Inflammatory Assays

The anti-inflammatory assays were performed based on previously published articles [23,29,30], under the aseptic conditions described in Section 2.6. The HEK-Blue™ hTLR2, HEK-Blue™ hTLR4, NOD2-WT, and NOD2-G908R cell lines were harvested when 90% confluent, and seeded at a density of 15,000 cells/well in 96-well plates. Following a 24 h incubation with the conditions described in Section 2.3, tomato extracts were added to the plate at gradient concentrations (17.86–95.24 mg dry tomato equivalent/mL) using a freshly prepared dosing plate. In addition, the positive control ibuprofen, negative control PMA, and solvent control DMSO were added to the cell plates by using the same dosing plate. The optimized ibuprofen concentration range was between 0.4–2.14 mM (82.40–440.84 μg/mL). After another 24 h incubation, 30 μL of ligand (or media for negative-ligand treatments) was added to each well of the well plates. The ligands used for stimulating the HEK-Blue™ hTLR2 and hTLR4 cells were 10 ng/mL of Pam3CSK4 and 3.125 μg/mL of LPS, respectively. NOD2-WT and NOD2-G908R cells were stimulated with 22.72 μg/mL of MDP. The plates were then incubated for another 24 h. 

An amount of 30 μL cell supernatant was transferred from each well of the 96-well plate into a new 96-well plate. Subsequently, 150 μL of QUANTI-Blue™ solution, prepared according to the instruction from the manufacturer, was added to each well. Following a 10 min incubation at 37 °C, the absorbance was measured at 650 nm by using a MultiSkan® Spectrum microplate spectrophotometer (ThermoScientific™, USA). Simultaneously, a rapid cytotoxicity screening on the four cell lines was also performed by using the WST-1 assay [23,29,30]. Briefly, 10 μL of WST-1 solution, prepared according to the instruction given by the manufacturer, was added to the remaining cell suspensions in the cell plate. Following a one hour incubation at 37 °C, the absorbance was measured at 450 nm. The cell viability (WST-1) and SEAP production (QUANTI-BLUE™) values were calculated and normalized against untreated cell and solvent controls, respectively. The anti-inflammatory experiments were performed in two biological repeats.

### 2.8. Data Analyses

The normality of the results of the antioxidant analyses was evaluated using the Shapiro-Wilk test performed using SPSS. The same software was then used to analyze the results using one-way analysis of variance (ANOVA) and Tukey’s honestly significant difference (HSD) post-hoc test. These statistical analyses were performed to test the differences between the antioxidant capacity of extracts from the same varieties, and subsequently the extracts with the highest antioxidant power from each variety. Pooled standard deviation (SD) was calculated using the formula given by Cohen [31].

The data obtained from the cell proliferation assay were used to generate a dose-response curve for each extract and cell line using Microsoft® Excel 2017 for Mac (Microsoft®, USA). Thereafter, the IC50 of each extract was determined by using the logit function of SPSS.

Analysis of the data from the anti-inflammatory assays was carried out based on Folkard et al. [23] and Peng et al. [30]. The WST-1 values were normalized to the untreated cell control and values <0.7 were considered to be cytotoxic. The SEAP production in HEK-Blue™ hTLR2 and hTLR4 cell lines were normalized relative to the ligand-positive solvent control to measure the anti-inflammatory activities of the extracts. Thereafter, a best-fit curve for the SEAP production was generated by using Microsoft Excel, and their IC30, IC50, and IC70 values were subsequently calculated using the logit function of SPSS. The SEAP production in NOD2 cells was normalized relative to the ligand-negative solvent control to measure both the ligand sensitivity of the cell lines and anti-inflammatory activities of the extracts. One-way ANOVA was subsequently used to measure the SEAP results from the NOD2 cells.

## 3. Results

The main objective of this study is to compare the in vitro biological activities of different tomato varieties. Consequently, the results of this study are presented as the dry weight equivalent of tomato, rather than weight of compounds. 

### 3.1. Antioxidant Assays

The results of the FRAP and ABTS assays are presented in Table 2. In terms of solvent, it was observed in both assays that the hexane extract of the four tomato varieties possessed significantly greater antioxidant activity than other extracts of the same varieties. It was then followed by the hexane-acetone (HA) extract, which had significantly higher antioxidant potential than the hexane-acetone-ethanol (HAE) and hexane-ethanol (HE) extracts. The antioxidant capacities of the HAE and HE extracts were significantly different in some varieties. 

A comparison of the antioxidant capacity of the varieties was performed using their respective hexane extracts as they possess superior antioxidant activities compared to extracts prepared from other solvents. The results of the FRAP assay reveals that the Olga’s Round Golden Chicken Egg has a lower antioxidant property than the other three varieties, which are not significantly different from each other. In addition, the ABTS assay also showed identical results apart from the fact that Golden Green was shown to possess significantly higher antioxidant power than that of the Alfred and Golden Eye. It is worth mentioning that although there was no statistical difference found in the FRAP results of Alfred, Golden Green, and Golden Eye, the trend showed that Golden Green had the highest antioxidant potential. Hence, these results imply that Golden Green has the highest in vitro antioxidant capacity compared to the other three varieties, while Olga’s Round Golden Chicken Egg has the least.

### 3.2. Anti-Proliferative Activity

**Figure 1:**
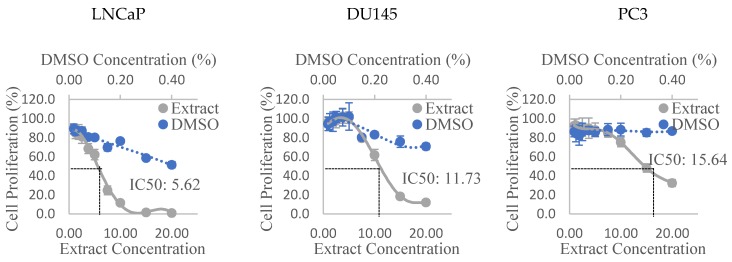
Dose-response curves of prostate cancer cell lines treated with the hexane extract of Olga’s Round Golden Chicken Egg tomato. Values are Mean ± Pooled SD; DMSO: Dimethyl sulfoxide; Extract concentrations and IC50 are expressed in mg dry tomato equivalent/mL.

The dose-response curves of the Olga’s Round Golden Chicken Egg hexane extract in all three PCa cell lines can be observed in Figure 1. Similar graphs of all other extracts were used to derive the IC50 of each extract, and the values are presented in Table 3. It can be seen that the Alfred HA, Olga’s Round Golden Chicken Egg hexane, and Golden Green hexane extracts were the most anti-proliferative extracts, amongst extracts of their respective varieties, to the three PCa cell lines. Additionally, the results also show that the Olga’s Round Golden Chicken Egg hexane extract had stronger anti-proliferative activities than any other extracts, followed by the Golden Green hexane extract, and subsequently Alfred HA extract. The extracts were shown to be most anti-proliferative to LNCaP cells, and least anti-proliferative to PC3 cells. Lastly, the results also show that extracts prepared from the Golden Eye variety appear to promote, rather than suppress cell proliferation in all three PCa cell lines.

### 3.3. Anti-Inflammatory Activity Mediated through the TLR2 and TLR4 Pathways

Based on the results of the antioxidant and anti-proliferative assays, we concluded that the HAE and HE extracts are less effective compared to the hexane and HA extracts. Hence, screening of the TLR2 and TLR4 anti-inflammatory activity was only performed on the hexane and HA extracts of the four tomato varieties.

The IC30, IC50, and IC70 of SEAP production in HEK-Blue™ hTLR2 and hTLR4 cell lines treated with tomato extracts are presented in Table 4. Additionally, the data from the positive control (ibuprofen) are also shown in the same Table. Our results show that the hexane extracts are generally less anti-inflammatory than the HA extracts of the same varieties. Furthermore, it was seen that the hexane extracts are more cytotoxic than the HA extracts, as depicted by some IC30, IC50, and IC70 concentrations that are cytotoxic in hexane extracts, but not in HA extracts.

Table 4 also shows that extracts prepared from the Golden Eye variety did not exhibit any meaningful anti-inflammatory activity. However, extracts prepared from Alfred, Olga’s Round Golden Chicken Egg, and Golden Green varieties were shown to possess anti-inflammatory activity to different extents. Based on the HA extracts, the highest anti-inflammatory activity was observed in Golden Green, followed by Olga’s Round Golden Chicken Egg, and subsequently Alfred.

### 3.4. NOD2-Mediated Anti-Inflammatory Pathway

The screening on TLR2 and TLR4-related anti-inflammatory pathways presented in the previous section revealed that the hexane extracts are less anti-inflammatory compared to the HA extracts. In addition, it was also observed that extracts prepared from the Golden Eye variety had undetectable effects on inflammation. Therefore, the screening on the NOD2 pathway was only performed on the HA extracts of the Alfred, Olga’s Round Golden Chicken, and Golden Green varieties.

**Figure 2:**
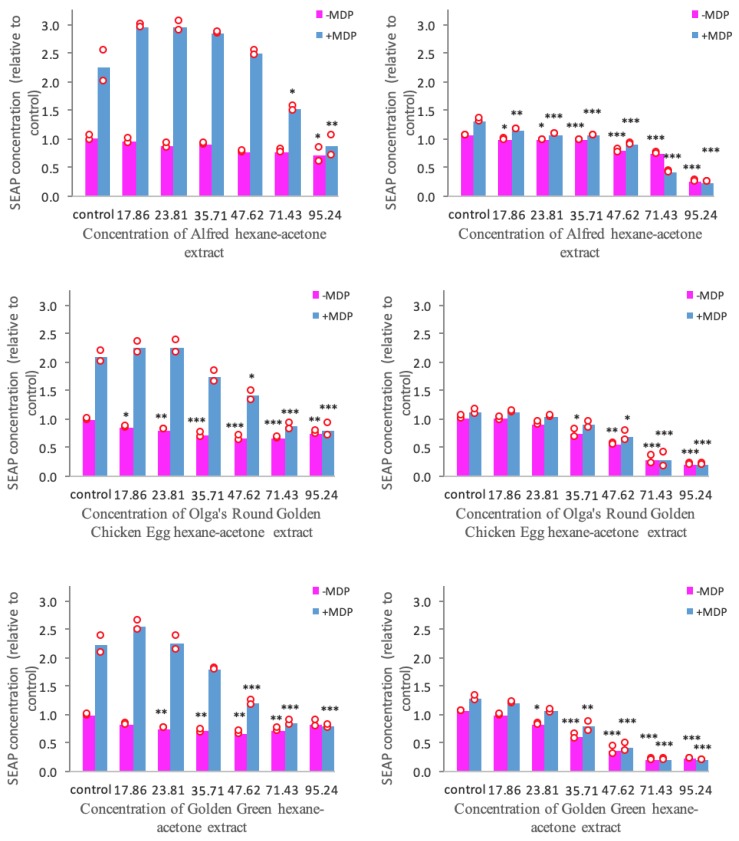
Normalized SEAP production in NOD2-WT (left) and NOD2-G908R (right) cell lines treated with three different tomato extracts. Bars represent Mean; Red circles depict the values obtained from each biological repeat; SEAP: Secreted embryonic alkaline phosphatase; MDP: muramyl dipeptide; Extract concentrations are expressed in mg dry tomato equivalent/mL; Statistical significance was reported against control; * indicates significant difference at *p* < 0.05; ** indicates significant difference at *p* < 0.01; *** indicates significant difference at *p* < 0.001.

The SEAP production in NOD2-WT and NOD2-G908R cell lines treated with tomato extracts is presented in Figure 2. It was observed that MDP stimulation initiates a 2 to 2.5-fold increase of SEAP production in NOD2-WT cells. Additionally, it was also seen that the NOD2-G908R cells are less sensitive to ligand stimulation compared to the NOD2-WT cells.

It can be seen from Figure 2 that the hexane-acetone extracts of the Alfred, Olga’s Round Golden Chicken Egg, and Golden Green varieties suppress NOD2-mediated inflammation to different extents. It can be seen that all three tomato extracts were able to suppress the MDP-stimulated SEAP production in NOD2-G908R cells at *p* < 0.001. However, only the Olga’s Round Golden Chicken Egg and Golden Green extracts were able to suppress the MDP-stimulated SEAP production in NOD2-WT cells at *p* < 0.001. 

Figure 2 also shows that all three tomato extracts were able to inhibit the unstimulated SEAP production in both NOD2-WT and NOD2-G908R cells to different degrees. Similar to the inhibition of the MDP-stimulated SEAP production, the extracts were able to inhibit the unstimulated SEAP production in NOD2-G908R cells at *p* < 0.001. Furthermore, the Olga’s Round Golden Chicken Egg and Golden Green extracts were also able to reduce the unstimulated SEAP production in NOD2-WT cells at *p* < 0.001.

In short, our results show that all three tomato extracts are promising candidates for suppressing NOD2-mediated inflammation. However, the Alfred extract was found to inhibit the SEAP production of NOD2-WT cells to a lesser extent than the other two extracts. Hence, it is fair to say that the Olga’s Round Golden Chicken Egg and Golden Green varieties have greater anti-inflammatory activity than the Alfred extracts through the NOD2-mediated pathway.

## 4. Discussion

### 4.1. Antioxidant Activity

In this study, the antioxidant capacities of the tomato extracts were screened through the FRAP and ABTS assays. Although it seemed somewhat redundant to perform multiple antioxidant activity measurements, it needs to be taken into consideration that each assay has different mechanisms, pH levels, redox potentials, reaction media, etc. [32]. Additionally, major organizations such as the International Union of Pure and Applied Chemistry (IUPAC) [32] and Association of Official Analytical Chemists (AOAC) [33] have concluded that there is no single antioxidant assay that can represent the antioxidant capacity of a sample. Thus, it was necessary to perform more than one antioxidant assay in order to carry out a reliable screening. The 2,2-diphenyl-1-picrylhydrazyl (DPPH), FRAP, and ABTS assays are considered as the most common in vitro antioxidant assays [34]. Nevertheless, the DPPH assay was not performed in this study as it has been reported that various carotenoids, including lycopene and beta-carotene, which are of interest in this study, have poor reactivity with the DPPH reagents [35].

The results of the FRAP and ABTS assessments (Table 2) imply that the Golden Green variety has the highest antioxidant potential among the four tomato varieties, while the Olga’s Round Golden Chicken Egg variety has the lowest. Since both varieties were reported to contain reasonably similar amounts of tetra-*cis* lycopene [36], these results imply that the in vitro antioxidant potential of the extracts is not attributed to tetra-*cis* lycopene alone. It is, therefore, unclear whether tangerine tomatoes possess greater or lower antioxidant activity compared to red tomatoes, and perhaps generalization should be avoided.

Ultimately, the results were obtained through in vitro chemical models which do not share similarities to biological systems within the human body [32]. Hence, in order to avoid exaggeration, the results were merely interpreted for an in vitro rapid screening, and no implications regarding the in vivo antioxidant activity of the examined extracts can be drawn. Based on the result of this in vitro screening, we therefore suggest a cell-based assessment (e.g. oxidative stress biomarker assay) to be performed on the hexane extracts of all four varieties. This will allow a better understanding of the potential effect of the tomato extracts on in vivo oxidative stress and its implication for oxidative-related diseases. 

### 4.2. Anti-Proliferative Activity

The results of the cell proliferation assay reveal that some extracts prepared from the Alfred, Olga’s Round Golden Chicken Egg, and Golden Green varieties were anti-proliferative to the PCa cell lines. However, the extracts prepared from the Golden Eye variety were shown to increase the proliferation of PCa cells. As the major constituent of the Golden Eye variety was identified as beta-carotene [36], it is implied that this pro-proliferative activity is associated with beta-carotene. This discovery is partially supported by the literature as there are various contradictory reports about the role of beta-carotene on cancer cell proliferation. Both Kotake-Nara et al. [37] and Williams et al. [38] found that the proliferation of PCa cell lines LNCaP, DU145, and PC3 was reduced in response to treatment with beta-carotene. However, Dulińska et al. [39] reported that beta-carotene supplementation was found to increase the proliferation of LNCaP cells. Nonetheless, both our research and the abovementioned studies were performed in vitro, the findings of which cannot be directly translated in vivo. With this in mind, the results of our in vitro experiment suggest that PCa patients should consume the Golden Eye, or other high-beta carotene foods in moderation, and further research is required.

Based on our results, it is reasonable to say that, in descending order, Olga’s Round Golden Chicken Egg, Golden Green, and Alfred varieties are promising candidates to for further assessment of their potential to enhance the treatment of or prevent PCa. Therefore, it is implied that tangerine tomatoes possess greater in vitro anti-proliferative activity to PCa cells than red or high-beta carotene tomatoes. 

### 4.3. Anti-Inflammatory Activity

Anti-inflammatory activities of the tomato extracts were assessed through the TLR2, TLR4, and NOD2-mediated inflammatory pathways. In addition to the fact that chronic inflammation is linked to cancer [14], single nucleotide polymorphisms (SNPs) of these genes have been associated with inflammatory bowel disease (IBD), a cancer-prone condition [40,41]. IBD has no elixirs, and various treatment options that are available, including glucocorticosteroids and aminosalicylates, have been identified to cause side effects [42]. Consequently, personalized dietary intervention is one of the potential IBD treatment options that are receiving increasing attention, since it is thought to cause minimal adverse effects [43,44]. Previous studies have shown that in vitro inflammation triggered through the aforementioned pathways might be alleviated by treatments with plant-based dietary bioactives [23,29,30]. Tomatoes have been shown to reduce the in vitro and in vivo expression of some pro-inflammatory cytokines [45,46]. Hence, tomatoes might have the potential to regulate inflammation initiated through the TLR2, TLR4, and NOD2-mediated pathways. To the best of our knowledge, this is the first study which investigates the effects of tomato extracts on these inflammatory pathways.

The HEK-Blue™ hTLR2, HEK-Blue™ hTLR4, and NOD2 cell lines are genetically modified cell lines, with limited pathways leading to NF-κB activation [22]. Hence, they can only be stimulated by the specific pattern recognition receptors (PRRs) that have been transfected in them, namely TLR2 for HEK-Blue™ hTLR2, TLR4 for HEK-Blue™ hTLR4, and NOD2 for NOD2-WT and NOD2-G908R. Hence, since the cell lines have been transfected with an NF-κB-inducible SEAP reporter, the SEAP production measured through the QUANTI-Blue™ assay reflects the stimulation of each PRR. In addition, the WST-1 cytotoxicity screening was performed because cell death may appear anti-inflammatory, and it is therefore important to perform this screening. This argument was evident in the hexane extracts of the Alfred (only in TLR2), Olga’s Round Golden Chicken Egg, and Golden Green varieties, where the IC30, IC50, and IC70 values of the extracts were found at cytotoxic concentrations (Table 4).

#### 4.3.1. TLR2 and TLR4 Pathways

The SEAP inhibition values of the positive control obtained in this study were similar to that reported by Peng et al. [30]. Hence, the results obtained (Table 4) can be interpreted with reasonable confidence. Although it is evident that the HA extracts of Alfred, Olga’s Round Golden Chicken Egg, and Golden Green tomatoes suppressed TLR2 and TLR4 induced inflammation, ibuprofen also showed similar effects at lower doses. This is, however, plausible as ibuprofen is a drug which is consumed in trace amounts, whilst tomatoes are foods which are consumed in larger amounts. Ultimately, from this screening, we can conclude that the Alfred, Olga’s Round Golden Chicken Egg, and Golden Green varieties might have the potential to be applied to the treatment of TLR2 and TLR4 associated IBD, whilst the Golden Eye variety does not appear to have this potential. Furthermore, the results imply that the tangerine tomatoes possess greater TLR2 and TLR4-facilitated anti-inflammatory activity than red and high-beta carotene tomatoes.

#### 4.3.2. NOD2 Pathway

It was seen that the NOD2-G908R was less sensitive to MDP-stimulation than the NOD2-WT, which is as expected [22,30]. This might have been caused by alterations in the NOD2 protein structure due to the presence of the SNP [47]. Apart from reducing the ligand sensitivity, the SNP is also thought to cause enhanced NF-κB activation due to the auto-activation of the NOD2 signaling cascade [47]. This might be the reason why the SNP is associated with IBD. From the results (Figure 2), it is evident that the Olga’s Round Golden Chicken Egg and Golden Green varieties have greater potential to be used in the treatment of NOD2-associated (WT and G908R SNP) IBD than the Alfred variety. Thus, it is implied that tangerine tomatoes high in tetra-*cis* lycopene have greater in vitro NOD2-mediated anti-inflammatory potential than red tomatoes.

### 4.4. Future Research

Based on our screening, tangerine tomatoes are shown to possess greater in vitro anticancer and anti-inflammatory activities than the red and high-beta carotene tomatoes. Nonetheless, it is unclear whether tangerine tomatoes possess greater or lower antioxidant power compared to the red and high-beta carotene tomatoes. In addition to the tetra-*cis* lycopene in tangerine tomatoes (Olga’s Round Golden Chicken Egg and Golden Green), all-*trans* lycopene in red tomatoes (Alfred), and beta-carotene in high beta-carotene tomatoes (Golden Eye), a potential bioactive present in the extracts may include lutein [36]. However, the possibilities are not limited to the abovementioned compounds as a study by Cooperstone et al. [48] reported the presence of phytoene, phytofluene, zeta-carotene, neurosporene, and various *cis* isomers of lycopene in tangerine tomatoes. Consequently, it is suggested that the identification and quantification of the major constituents of each tomato variety be carried out using analytical methods, such as the high-performance liquid chromatography-diode array detector-tandem mass spectrophotometry (HPLC-DAD-MS/MS). The bioactivities of these constituents can then be screened to determine the compound responsible for the biological activities of the extract, as well as their synergistic activities. Furthermore, for the cell-based assays, the concentration of the specific constituents in the treated medium should be determined prior to and after the incubation period. This will allow a better understanding of the uptake of those constituents by the cells.

An additional suggestion for future research would be for in vivo studies to be performed. This will serve as a validation to the anti-proliferative and anti-inflammatory activities observed. Since both cancer and IBD have no universal cure, it is worth investigating whether dietary components like tomatoes can alleviate these chronic diseases or reduce their progression in vivo. Furthermore, since tetra-*cis* lycopene in tangerine tomatoes has been reported to be more bioavailable than their all-*trans* counterpart [7,8,9], research on their in vivo health benefits is of interest. Ultimately, since it has been reported that tetra-*cis* lycopene is not heat stable [48], it is suggested that in vivo studies be performed on raw or minimally thermal-processed tangerine tomatoes.

## 5. Conclusions

The Alfred, Olga’s Round Golden Chicken Egg, Golden Green, and Golden Eye tomato varieties were all shown to possess in vitro antioxidant activity through the FRAP and ABTS assays. Nonetheless, only Alfred, Olga’s Round Golden Chicken Egg, and Golden Green were shown to have in vitro anti-proliferative and anti-inflammatory activities. Among the extraction solvents used, hexane and HA were determined to be the solvents which produced extracts with the highest biological activities. Specifically, the hexane extracts of the four varieties were determined to be the extracts with the highest antioxidant capacity among extracts of their respective varieties. Furthermore, Olga’s Round Golden Chicken Egg hexane, Golden Green hexane, and Alfred HA were, in descending order, determined to be the most anti-proliferative extracts to the LNCaP, DU145, and PC3 cell lines. Moreover, the HA extracts of Golden Green, Olga’s Round Golden Chicken Egg, and Alfred were, in descending order, found to be the most anti-inflammatory extracts through the TLR2, TLR4, and NOD2 pathways. These findings imply that tomatoes, particularly the tangerine tomatoes (Olga’s Round Golden Chicken Egg and Golden Green) and to a lesser extent the red (Alfred) tomatoes, have the potential to be further studied for their roles in the treatment and prevention of chronic diseases, namely PCa and IBD. Nevertheless, this conclusion is premature and will need an in vivo study as a form of validation.

## Figures and Tables

**Table 1:** List of extraction solvents utilized in this study.

Solvent	Ratio	Source
Hexane	-	[17]
Hexane:Acetone:Ethanol	2:1:1	[18]
Hexane:Acetone	1:1	[19]
Hexane:Ethanol	3:4	[20]

**Table 2:** Antioxidant activity of tomato extracts as assessed through the FRAP and ABTS assays.

Extraction Solvent	Tomato Variety			
	Alfred	Olga’s Round Golden Chicken Egg	Golden Green	Golden Eye
FRAP Assay				
Hexane	11.71 ± 0.62 ^d/B^	6.24 ± 0.30 ^c/A^	12.52 ± 0.54 ^c/B^	12.50 ± 0.35 ^d/B^
HAE	3.66 ± 0.12 ^b^	2.23 ± 0.21 ^a^	5.99 ± 0.31 ^a^	6.18 ± 0.34 ^b^
HA	7.10 ± 0.45 ^c^	4.72 ± 0.36 ^b^	10.54 ± 0.17 ^b^	8.60 ± 0.54 ^c^
HE	2.11 ± 0.22 ^a^	1.79 ± 0.22 ^a^	5.58 ± 0.25 ^a^	3.23 ± 0.23 ^a^
ABTS Assay				
Hexane	20.04 ± 1.40 ^d/B^	14.27 ± 1.03 ^d/A^	26.22 ± 0.32 ^c/C^	18.32 ± 1.16 ^d/B^
HAE	6.23 ± 0.50 ^b^	6.82 ± 0.64 ^b^	14.42 ± 1.06 ^a^	8.24 ± 0.70 ^b^
HA	13.84 ± 0.33 ^c^	11.46 ± 1.16 ^c^	19.76 ± 1.60 ^b^	11.87 ± 0.69 ^c^
HE	1.99 ± 0.20 ^a^	6.11 ± 0.44 ^a^	13.42 ± 1.04 ^a^	3.77 ± 0.26 ^a^

Results are expressed as Mean ± Pooled SD; Results are expressed in mmol α-tocopherol/g dry tomato equivalent; HAE: hexane-acetone-ethanol; HA: hexane-acetone; HE: hexane-ethanol; Different lowercase superscript indicates significant difference at *p* < 0.05 compared to other values in the same column and assay; Different UPPERCASE superscript indicates significant difference at *p* < 0.05 compared to other values in the same row and assay.

**Table 3:** IC50 of three prostate cancer cell lines treated with different tomato extracts.

Tomato Variety	Extraction Solvent			
	Hexane	HAE	HA	HE
LNCaP				
Alfred	14.46	X	11.76†	X
Olga’s	**5.62 †**	16.93	10.1	X
Golden Green	8.08 †	15.45	10.19	X
Golden Eye	+	+	+	+
DU145				
Alfred	X	X	-†	X
Olga’s	**11.73 †**	X	-	X
Golden Green	17.84 †	X	18.01	X
Golden Eye	+	+	+	+
PC3				
Alfred	X	X	-†	X
Olga’s	**15.64 †**	X	-	X
Golden Green	-†	X	X	X
Golden Eye	+	+	+	+

Results are expressed in mg dry tomato equivalent/mL; Olga’s: Olga’s Round Golden Chicken Egg; HAE: hexane-acetone-ethanol; HA: hexane-acetone; HE: hexane-ethanol; + indicates a pro-proliferative effect was observed relative to solvent control; - indicates anti-proliferative effect was observed relative to solvent control, but IC50 was not achieved; X indicates that no effect on cell proliferation was observed relative to solvent control; † indicates the extract that is more anti-proliferative than other extracts from the same variety (same row), to a specific cell line; **bold** indicates the most-anti-proliferative extract relative to all sixteen extracts tested on each cell line.

**Table 4:** Inhibition of SEAP production in HEK-Blue™ cells following treatment with tomato extracts and ibuprofen.

-	Concentration (mg Dry Tomato Equivalent/mL)								Ibuprofen (mg/mL)
	Alfred Hexane	Alfred HA	Olga’s Hexane	Olga’s HA	GG Hexane	GG HA	GE Hexane	GE HA	
HEK-Blue™ hTLR2									
IC30	60.24	59.35	53.69 *	39.96	50.01 *	32.83	N/A	N/A	0.27
IC50	88.46	74.73	66.22 *	55.62	61.16 *	50.41	N/A	N/A	0.4
IC70	N/A	90.1	78.75 *	71.28	72.3 *	67.99	N/A	N/A	N/A
HEK-Blue™ hTLR4									
IC30	84.56 *	64.47	52.2 *	51.51	56.69 *	42.76	N/A	N/A	0.15
IC50	N/A	76.01	61.44 *	62.53	62.89 *	53.08	N/A	N/A	0.27
IC70	N/A	87.55	70.68 *	73.55	69.09 *	63.4	N/A	N/A	0.38

HA: hexane-acetone; Olga’s: Olga’s Round Golden Chicken Egg; GG: Golden Green; GE: Golden Eye; * denotes the doses determined to be toxic through the WST-1 cytotoxicity screening (data not shown); N/A: not achieved within the concentration ranged applied in this study.
